# Rapidly progressive interstitial lung disease risk prediction in anti-MDA5 positive dermatomyositis: the CROSS model

**DOI:** 10.3389/fimmu.2024.1286973

**Published:** 2024-02-01

**Authors:** Lei Wang, Chengyin Lv, Hanxiao You, Lingxiao Xu, Fenghong Yuan, Ju Li, Min Wu, Shiliang Zhou, Zhanyun Da, Jie Qian, Hua Wei, Wei Yan, Lei Zhou, Yan Wang, Songlou Yin, Dongmei Zhou, Jian Wu, Yan Lu, Dinglei Su, Zhichun Liu, Lin Liu, Longxin Ma, Xiaoyan Xu, Yinshan Zang, Huijie Liu, Tianli Ren, Jin Liu, Fang Wang, Miaojia Zhang, Wenfeng Tan

**Affiliations:** ^1^ Division of Rheumatology, The First Affiliated Hospital of Nanjing Medical University, Nanjing, Jiangsu, China; ^2^ Division of Rheumatology, Wuxi People’s Hospital, Wuxi, Jiangsu, China; ^3^ Division of Rheumatology, Huai’an First People’s Hospital, Huai’an, Jiangsu, China; ^4^ Division of Rheumatology, The First People’s Hospital of Changzhou, Changzhou, Jiangsu, China; ^5^ Division of Rheumatology, Affiliated Hospital of Nantong University, Nantong, Jiangsu, China; ^6^ Division of Rheumatology, Northern Jiangsu People’s Hospital, Yangzhou, Jiangsu, China; ^7^ Division of Rheumatology, Changzhou No.2 People’s Hospital, Changzhou, Jiangsu, China; ^8^ Division of Rheumatology, Affiliated Hospital of Xuzhou Medical University, Xuzhou, Jiangsu, China; ^9^ Division of Rheumatology, The First Affiliated Hospital of Soochow University, Suzhou, Jiangsu, China; ^10^ Division of Rheumatology, Jiangsu Province Hospital of Chinese Medicine, Nanjing, Jiangsu, China; ^11^ Division of Rheumatology, Nanjing First Hospital, Nanjing, Jiangsu, China; ^12^ Division of Rheumatology, The Second Affiliated Hospital of Soochow University, Suzhou, Jiangsu, China; ^13^ Division of Rheumatology, Xuzhou Central Hospital, Xuzhou, Jiangsu, China; ^14^ Division of Rheumatology, Yancheng No.1 People’s Hospital, Yancheng, Jiangsu, China; ^15^ Division of Rheumatology, Zhongda Hospital Southeast University, Nanjing, Jiangsu, China; ^16^ Division of Rheumatology, The Affiliated Suqian First People’s Hospital of Nanjing Medical University, Suqian, Jiangsu, China; ^17^ Division of Rheumatology, The First People’s Hospital of Lianyungang, Lianyungang, Jiangsu, China; ^18^ Division of Rheumatology, Wuxi No.2 People’s Hospital, Wuxi, Jiangsu, China; ^19^ Research Institute of Clinical Medicine, The First Affiliated Hospital of Nanjing Medical University, Nanjing, Jiangsu, China; ^20^ Division of Cardiology, The First Affiliated Hospital of Nanjing Medical University, Nanjing, Jiangsu, China

**Keywords:** anti-melanoma differentiation-associated gene 5, dermatomyositis, rapidly progressive interstitial lung disease, predict models, easy-to-use

## Abstract

**Background:**

The prognosis of anti-melanoma differentiation-associated gene 5 positive dermatomyositis (anti-MDA5^+^DM) is poor and heterogeneous. Rapidly progressive interstitial lung disease (RP-ILD) is these patients’ leading cause of death. We sought to develop prediction models for RP-ILD risk in anti-MDA5^+^DM patients.

**Methods:**

Patients with anti-MDA5^+^DM were enrolled in two cohorts: 170 patients from the southern region of Jiangsu province (discovery cohort) and 85 patients from the northern region of Jiangsu province (validation cohort). Cox proportional hazards models were used to identify risk factors of RP-ILD. RP-ILD risk prediction models were developed and validated by testing every independent prognostic risk factor derived from the Cox model.

**Results:**

There are no significant differences in baseline clinical parameters and prognosis between discovery and validation cohorts. Among all 255 anti-MDA5^+^DM patients, with a median follow-up of 12 months, the incidence of RP-ILD was 36.86%. Using the discovery cohort, four variables were included in the final risk prediction model for RP-ILD: C-reactive protein (CRP) levels, anti-Ro52 antibody positivity, short disease duration, and male sex. A point scoring system was used to classify anti-MDA5^+^DM patients into moderate, high, and very high risk of RP-ILD. After one-year follow-up, the incidence of RP-ILD in the very high risk group was 71.3% and 85.71%, significantly higher than those in the high-risk group (35.19%, 41.69%) and moderate-risk group (9.54%, 6.67%) in both cohorts.

**Conclusions:**

The CROSS model is an easy-to-use prediction classification system for RP-ILD risk in anti-MDA5^+^DM patients. It has great application prospect in disease management.

## 1 Background

Anti-melanoma differentiation-associated gene 5 positive (anti-MDA5^+^) dermatomyositis (DM) is a subtype of DM characterized by distinct cutaneous lesions, little or no muscle involvement, and interstitial lung disease (ILD) (1). ILD is the most important clinical feature of anti-MDA5^+^DM affecting approximately 60–100% of patients; importantly, over 40% (20–75%) of them tend to develop the rapidly progressive ILD phenotype (RP-ILD), especially in the Asian population (2–5). Anti-MDA5^+^DM patients with RP-ILD are generally resistant to glucocorticoid and immunosuppressant therapy, leading to a 6-month mortality rate as high as 50-70% (6). Risk stratification to predict patients who will develop fatal RP-ILD at the early stage of the disease is very important for discussing patient expectations and supporting therapy decision-making in anti-MDA5^+^DM patients.

In the past decade, a great effort has been attempted to identify risk factors that predict ILD progression and mortality in anti-MDA5^+^DM. Old age, skin ulceration, and lack of myositis are suggested as risk factors for RP-ILD (7). Elevated serum CRP, ferritin level, and Krebs von den Lungen-6 (KL-6) levels have been linked to poor outcomes in RP-ILD patients (8, 9). Most recently, forced vital capacity has been validated as a predictor for the survival of ILD in anti-MDA5^+^DM (10). However, the accuracy of this single risk factor for prognosis estimation is limited by small size, lack of validation, and disease heterogeneity, hindering them from being genuinely applied in the clinic.

Recently, a clinical prediction model of FLAIR score that combines five clinical items, including ferritin, lactate dehydrogenase, anti-MDA5 antibody, CT imaging score, and RP-ILD, has been developed (11). The FLAIR risk score model could help to predict survival in amyopathic DM with ILD. However, the availability of certain items, such as the HRCT scoring system and anti-MDA5 antibody titers, limited the FLAIR model from being quickly adopted in daily clinical practice (12–14).

The current study aims to establish a simple-to-use risk prediction model for RP-ILD based on easily available clinical variables. Using a discovery cohort and an independent validation cohort, we identified and validated that the CROSS model is a readily available risk classification system for predicting RP-ILD in anti-MDA5^+^DM patients.

## 2 Methods

### 2.1 Study cohort and participants

**Figure 1:**
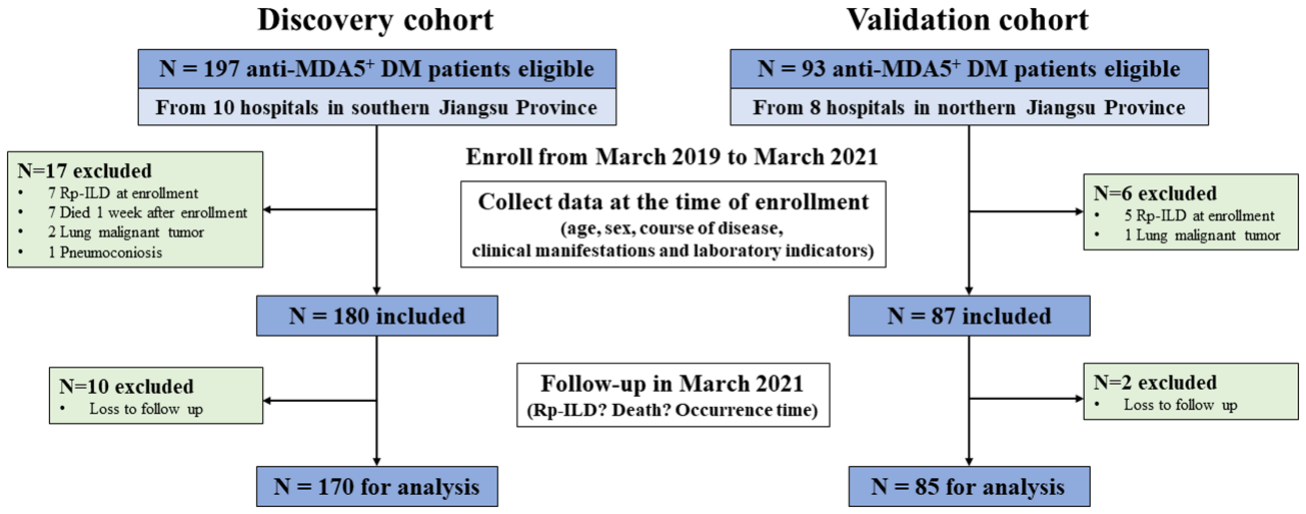
Flow chart of study enrollment. Patients who were diagnosed with dermatomyositis and had positive serum anti-MDA5 antibodies were included in this study. Among them, patients who presented with RP-ILD at enrollment and died within one week of enrollment, as well as those with lung malignant tumor, pneumoconiosis, and loss to follow-up were excluded. Anti-MDA5^+^DM, anti-melanoma differentiation-associated gene 5 positive dermatomyositis; RP-ILD, rapidly progressive interstitial lung disease.

Our cohort study was registered (ClinicalTrials.gov NCT04747652). Adult patients with DM meeting the European NeuroMusclar Center (ENMC) criteria or Sontheimer criteria were recruited into the cohort after excluding other autoimmune diseases or other causes of ILD (15, 16). Consecutive anti-MDA5^+^DM collected from ten tertiary hospitals in southern Jiangsu province from March 2019 to March 2021 were included in the discovery cohort. To externally verify the derived prediction model, 85 eligible consecutive anti-MDA5^+^DM patients were recruited into the validation cohort from eight tertiary hospitals in the northern region of Jiangsu province between March 2019 and March 2021 (**Figure 1**).

All clinical data were collected at baseline (initially diagnosed as DM), to death or the last follow-up visit. Clinical parameters of all subjects include the demographic information (including age at onset, gender, disease duration (defined as from disease onset to the cohort enrollment), initial symptoms associated with the disease), clinical manifestation (including muscle weakness, rash, periungual erythema, arthritis, mechanic’s hand, skin ulcer, and interstitial lung disease) and laboratory indicators (including alanine transaminase [ALT], aspartate aminotransferase [AST], lactic dehydrogenase [LDH], creatine kinase [CK], erythrocyte sedimentation rate [ESR], C-reactive protein [CRP], serum ferritin [SF], autoantibodies [including MDA5, ANA and Ro-52]). CT scans were obtained at 1- to 3-month intervals. Two expert thoracic radiologists re-reviewed all available HRCT imaging from baseline to death or the last follow-up visit.

The definition of RP-ILD was according to the descriptions in previous literature with some modifications (17, 18). Briefly, RP-ILD was defined as the presence of any of the following four conditions within one month after the onset of respiratory symptoms: 1) acute and progressive worsening of dyspnea requiring hospitalization or supplementary oxygen; 2) lung function including forced vital capacity (FVC) decreases by more than 10%, or diffusion capacity for carbon monoxide of the Lung (DLCO) falls over 15% with the decreased FVC; 3) high-resolution CT (HRCT) of the chest demonstrates that the extent of interstitial abnormalities increased more than 20%; 4) arterial blood gas analysis suggests respiratory failure or the oxygen partial pressure reduction is more significant than 10mmHg.

### 2.2 Statistical strategy and modeling

Medical records were reviewed retrospectively to collect clinical, laboratory, and imaging data, and all data were collected using an Electronic Data Capture (EDC) System explicitly designed for the cohort. If a patient developed RP-ILD during follow-up, it will be recorded as an endpoint event for poor outcomes.

In data processing, some continuous variables were transformed into dichotomies. Patients’ median age and disease duration at baseline were used as the cutoff value to distinguish elderly patients and short course of disease. The abnormal thresholds of laboratory indicators used the upper end of their normal reference range. Anti-MDA5 antibodies were tested by immunoblot testing (Euroimmun, Lubeck, Germany) in the same central laboratory for all subjects, and the high titer positivity is defined as +++. ANA is detected using the indirect immunofluorescence assay, and a titre greater than 1:40 is considered positive.

SPSS 23.0, GraphPad Prism 8.4.2, and R version 3.6.3 were used in the statistical analysis. Differences between the different groups (whether an RP-ILD and death endpoint event occurred in the discovery cohort) were calculated with the Mann-Whitney U test and Pearson’s chi-square test in measurement and categorical data, respectively. *P* < 0.05 was considered statistically significant, and all statistical tests were two-tailed probability tests.

A discovery cohort was used to develop a prediction model of RP-ILD in anti-MDA5^+^DM patients. All transformed dichotomous variables were tested by univariate Cox analysis to screen whether it is a related factor to the occurrence of RP-ILD. Some continuous variables were dichotomized based on the median, including age, course of the disease (short disease duration is defined as ≤ 3 months after anti-MDA5^+^DM onset), follow-up time, etc. In contrast, other continuous variables were dichotomized based on the upper limit of 95% confidence interval of clinical tests, including ALT, AST, LDH, CK, ESR, CRP and SF. Univariate and multivariable Cox analyses were then performed to identify the independent risk factors for developing RP-ILD in anti-MDA5^+^DM patients. Variables with p < 0.2 in the univariate analysis were included in the multivariable Cox analysis as the covariates. To identify independent prognostic risk factors and calculate their weightiness, β regression coefficient and integer estimation were used to form an integral model for predicting the occurrence of RP-ILD.

Time-dependent receiver operating characteristic (ROC) curve analysis was used to determine whether the two score models were the optimal clinical significance threshold. Kaplan-Meier method was used to calculate the cumulative poor prognosis rates during follow-up, and the logarithmic rank test was used to compare different risk groups. Finally, the incidence of RP-ILD and mortality at 3 months, 6 months, and 1 year in anti-MDA5^+^DM patients with varying levels of risk was accurately measured and demonstrated. The above verification methods are carried out in the external validation cohort to verify the prediction models’ reliability further.

## 3 Results

### 3.1 Baseline characteristics of anti-MDA5^+^DM patients in the discovery and validation cohort

**Table 1** shows the baseline characteristics of the discovery and validation cohorts. Patients in the discovery and validation cohorts do not show marked differences at baseline. As one group, anti-MDA5^+^DM patients are predominantly women (69.8%) with a median age of 53.0 (47.0-63.0) years old. The median follow-up time is 10.0 (3.0-14.0) and 12.0 (3.0-14.0) months in the discovery and validation cohorts, respectively. The overall incidence of RP-ILD in anti-MDA5^+^DM patients was 36.86% (94/255), and the mortality was 24.71% (63/255).

**Table 1:** Comparison of clinical manifestations and laboratory features between discovery and validation cohorts at baseline.

Parameters	Total (n=255)	Discovery Cohort (n=170)	Validation Cohort (n=85)	p value
General information				
Gender, male sex, No. (%)	77 (30.2%)	54 (31.8%)	23 (27.1%)	0.440
Age, median (Q1-Q3), years	53.0 (47.0-63.0)	53.0 (46.8-62.0)	53.0 (47.0-64.0)	0.568
Disease durations, median (Q1-Q3), months	3.0 (1.0-5.0)	3.0 (1.0-5.0)	3.0 (1.0-5.0)	0.482
Follow-up periods, median (Q1-Q3), months	12.0 (3.0-14.0)	10.0 (3.0-14.0)	12.0 (3.0-14.0)	0.165
Clinical manifestations				
Muscle weakness, No. (%)	117 (45.9%)	81 (47.7%)	36 (42.4%)	0.424
Rash, No. (%)	238 (93.3%)	162 (95.3%)	76 (89.4%)	0.076
Gottron’s sign, No. (%)	170 (66.7%)	113 (66.5%)	57 (67.1%)	0.925
Heliotrope rash, No. (%)	147 (57.7%)	104 (61.2%)	43 (50.6%)	0.107
V sign, No. (%)	92 (36.1%)	59 (34.7%)	33 (38.8%)	0.519
Shawl sign, No. (%)	59 (23.1%)	43 (25.3%)	16 (18.8%)	0.248
Periungual erythema, No. (%)	55 (21.6%)	36 (21.2%)	19 (22.4%)	0.830
Arthritis, No. (%)	92 (36.1%)	64 (37.7%)	28 (32.9%)	0.461
Mechanic’s hand, No. (%)	70 (27.5%)	41 (24.1%)	29 (34.1%)	0.092
Skin ulcer, No. (%)	36 (14.1%)	24 (14.1%)	12 (14.1%)	1.000
Laboratory features				
ALT, median (Q1-Q3), units/L	46 (28.5-85.0)	46.1 (28.8-79.1)	46 (28-95.4)	0.961
AST, median (Q1-Q3), units/L	52 (33.1-83)	53 (34-82.9)	50 (30–83)	0.429
LDH, median (Q1-Q3), units/L	340 (256–430)	340 (267-423.5)	337 (224-490.25)	0.895
CK, median (Q1-Q3), units/L	61.5 (36-144.3)	64 (36.5-141)	57 (34-163.5)	0.996
ESR, median (Q1-Q3), mm/H	37.1 (23–56)	39 (23-56.8)	37 (21–56)	0.741
CRP, median (Q1-Q3), mg/L	5.8 (3.1-12.3)	6.1 (3.1-14.5)	4.6 (2-10.8)	0.063
SF, median (Q1-Q3), ng/mL	869.5 (340.8-1500)	869.5 (323.8-1642.3)	886.3 (390.6-1500)	0.917
ANA, positive, No. (%)	131 (51.4%)	83 (48.8%)	48 (56.5%)	0.249
Anti-Ro52 antibody, positive, No. (%)	164 (64.3%)	114 (67.1%)	50 (58.8%)	0.196
Anti-MDA5 antibody, No. (%)				0.456
Low titer, +	76 (29.8%)	54 (31.8%)	22 (25.9%)	
Moderate titer, ++	46 (18.0%)	32 (18.8%)	14 (16.5%)	
High titer, +++	133 (52.2%)	84 (49.4%)	49 (57.7%)	
Prognosis				
RP-ILD, No. (%)	94 (36.9%)	60 (35.3%)	34 (40.0%)	0.463
3-months incidence rate	85/244 (34.8%)	51/160 (31.9%)	34/84 (40.5%)	0.180
6-months incidence rate	92/235 (39.2%)	58/151 (38.4%)	34/84 (40.5%)	0.756
12-months incidence rate	94/205 (45.9%)	60/122 (49.2%)	34/83 (41.0%)	0.246
Mortality, No. (%)	63 (24.7%)	42 (24.7%)	21 (24.7%)	1.000
3-months mortality	44/240 (18.3%)	25/157 (15.9%)	19/83 (22.9%)	0.185
6-months mortality	54/224 (24.1%)	33/144 (22.9%)	21/80 (26.3%)	0.576
12-months mortality	62/197 (31.5%)	41/118 (34.8%)	21/79 (26.68%)	0.227

*Data are presented as median (1st quartile [Q1]-3rd quartile [Q3]) or case number (percentage). Mann-Whitney U and Pearson’s Chi-square test were used to analysis. RP-ILD, rapidly progressive interstitial lung disease; ALT, alanine transaminase; AST, aspartate aminotransferase; LDH, lactic dehydrogenase; CK, creatine kinase; ESR, erythrocyte sedimentation rate; CRP, C-reactive protein; SF, serum ferritin; ANA, anti-nuclear antibodies; MDA5, anti-melanoma differentiation-associated gene 5.

### 3.2 Development of the CROSS model to predict the risk of RP-ILD

After univariate Cox analysis, variables with *p* < 0.2 were included in the multivariable Cox analysis as the covariates, including male sex, short disease duration, abnormal laboratory parameters (including AST, LDH, CK, CRP, and SF), anti-Ro52 antibody positivity and high titer positivity of anti-MDA5 antibody defined as +++.

**Figure 2:**
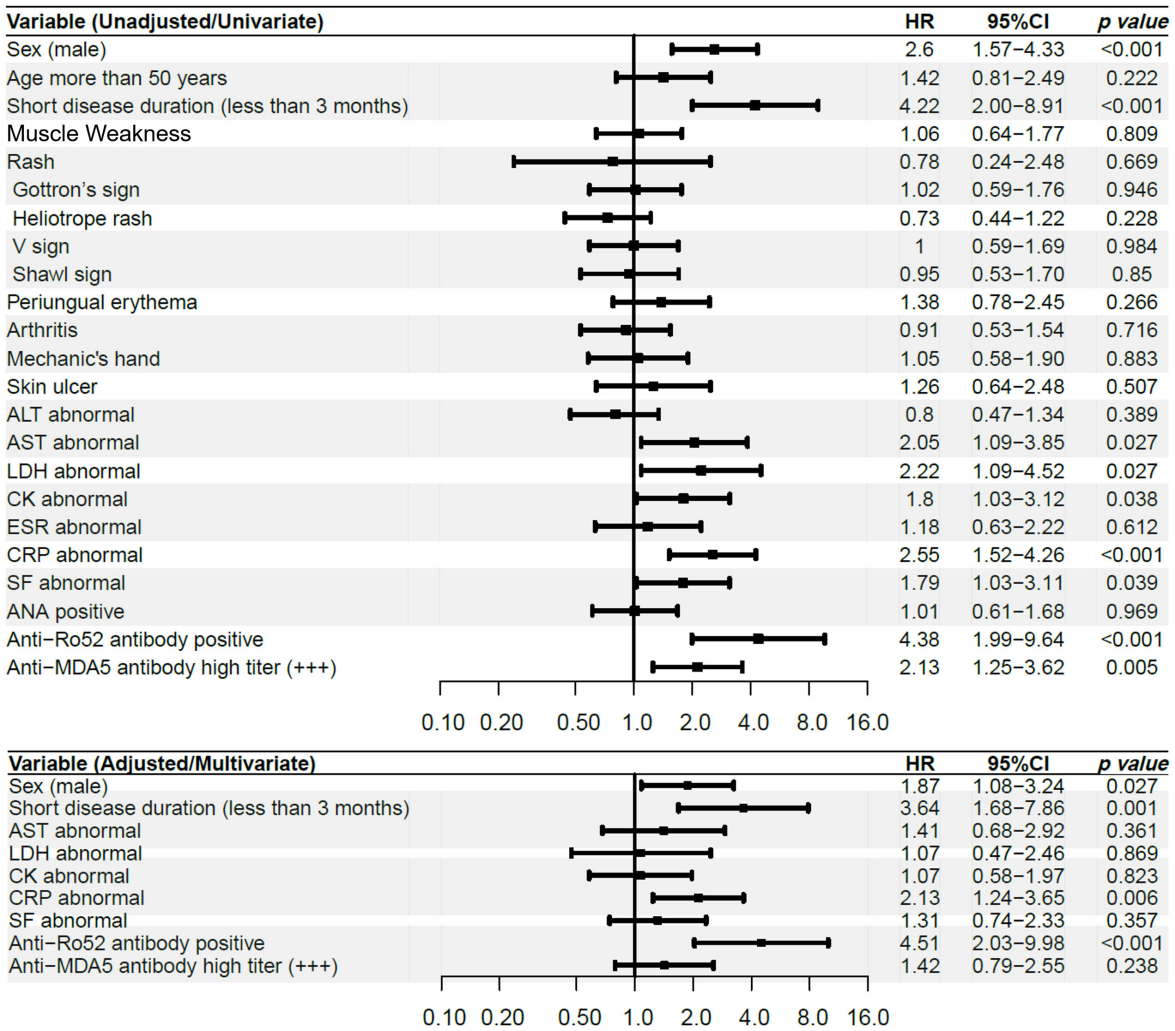
Univariate and Multivariate COX analysis of RP-ILD influenced by baseline manifestations in the discovery cohort **(A)** Univariate COX analysis, **(B)** Multivariate COX analysis. ALT, alanine transaminase; AST, aspartate aminotransferase; LDH, lactic dehydrogenase; CK= creatine kinase; ESR, erythrocyte sedimentation rate; CRP, C-reactive protein; SF, serum ferritin; ANA, anti-nuclear antibodies; MDA5, anti-melanoma differentiation-associated gene 5; RP-ILD, rapidly progressive interstitial lung disease; HR, Hazard ratio.

After COX regression, four risk factors were finally determined as the independent risk factors for the development of RP-ILD, including CRP abnormal (defined as exceed the upper limit of the normal detection range), anti-Ro52 antibody positivity, male sex, and short disease duration (**Figure 2**). We then selected these four independent risk factors to create a new RP-ILD risk prediction model of the CROSS score (CRP abnormal, anti-Ro52 antibody, sex [male sex], and short disease duration). These four variables were weighted according to the ratio of the β coefficient. When calculating the CROSS score, anti-Ro52 positivity and short disease duration scored 2 points, respectively, and abnormal CRP and male sex scored one point. The Alignment Diagram also shows the weighted relationship between these four predictors and the occurrence of RP-ILD, and the concordance index reached 0.825 (**Supplementary Figure 1**).

Based on the CROSS score with a range from 0 to 6, anti-MDA5^+^DM patients were classified as moderate risk (CROSS score = 0-2), high risk (CROSS score = 3-4), and very high risk (CROSS score = 5-6) for developing of RP-ILD, respectively (**Table 2**).

**Table 2:** CROSS prognostic score system in discovery cohort.

Parameters	β coefficient	Score
CRP abnormal	0.754	1
Anti-Ro52 positivity	1.505	2
Sex (male sex)	0.624	1
Short disease duration (less than 3 months)	1.291	2

†To predict the risk of RP-ILD, CROSS model: score 0-2, moderate risk; score 3-4, high risk; score 5-6 very high risk. CRP, C-reactive protein; AST, aspartate aminotransferase; RP-ILD, rapidly progressive interstitial lung disease; HR, Hazard ratio.

### 3.3 Efficiency validation of the CROSS model

To verify the accuracy of the predictive model, the CROSS score was calculated in both cohorts as the internal validation and external validation datasets, respectively. Compared with patients without RP-ILD, the CROSS score at baseline was significantly higher in anti-MDA5^+^DM patients with RP-ILD in both cohorts (**Supplementary Figure 2**). Then, time-dependent ROC curves were performed to evaluate the forecast effects of the CROSS model. The area under the curve (AUC) of the CROSS model in both cohorts are more than 0.8 at each time point within one year, which indicates excellent differentiation efficiency of the CROSS model (**Supplementary Figure 3**).

**Figure 3:**
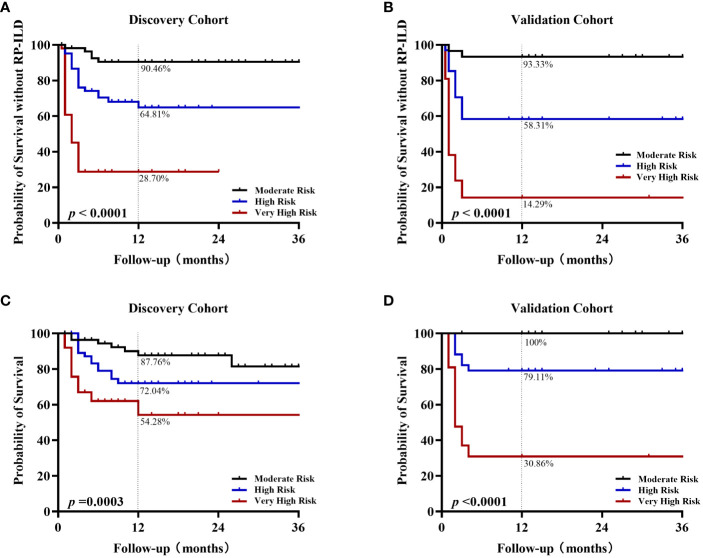
Kaplan-Meier analysis of different risk grades in survival rate and RP-ILD-free survival rate Kaplan-Meier non-RPILD survival curves for the different risk stratification groups according to the CROSS model in the discovery cohort **(A)** or validation cohort **(B)**. Kaplan-Meier survival curves in the discovery cohort **(C)** or validation cohort **(D)**.

Next, Kaplan-Meier analysis was used to assess whether the percentage of adverse events in patients at different risk levels differed significantly over time. The proportion of anti-MDA5^+^DM patients developing RP-ILD over time increases considerably as the risk level assessed based on the CROSS model increases in both cohorts (p< 0.0001). In the discovery cohort, the 1-year non-RP-ILD survival rate of the moderate-, high-, and very high-risk groups were 90.46%, 64.81%, and 28.7%, respectively (**Figure 3A**). In the validation cohort, the 1-year non-RP-ILD survival rate of the moderate-, high-, and very high-risk groups were 93.33%, 58.31%, and 14.29%, respectively (p< 0.0001) (**Figure 3B**). At the same time, we further analyzed the time-dependent mortality of these three groups of anti-MDA5^+^DM patients. The prognostic grouping based on the CROSS model also has an excellent predictive effect on the risk of death (p=0.0003 in the discovery cohort and p< 0.0001 in the validation cohort). The one-year survival rates of very high-risk patients were 54.28% and 30.86%, significantly lower than those in moderate- and high-risk patients (**Figures 3C, D**).

### 3.4 Incidence rate of poor prognosis in various risk grades evaluated by the models

**Figure 4:**
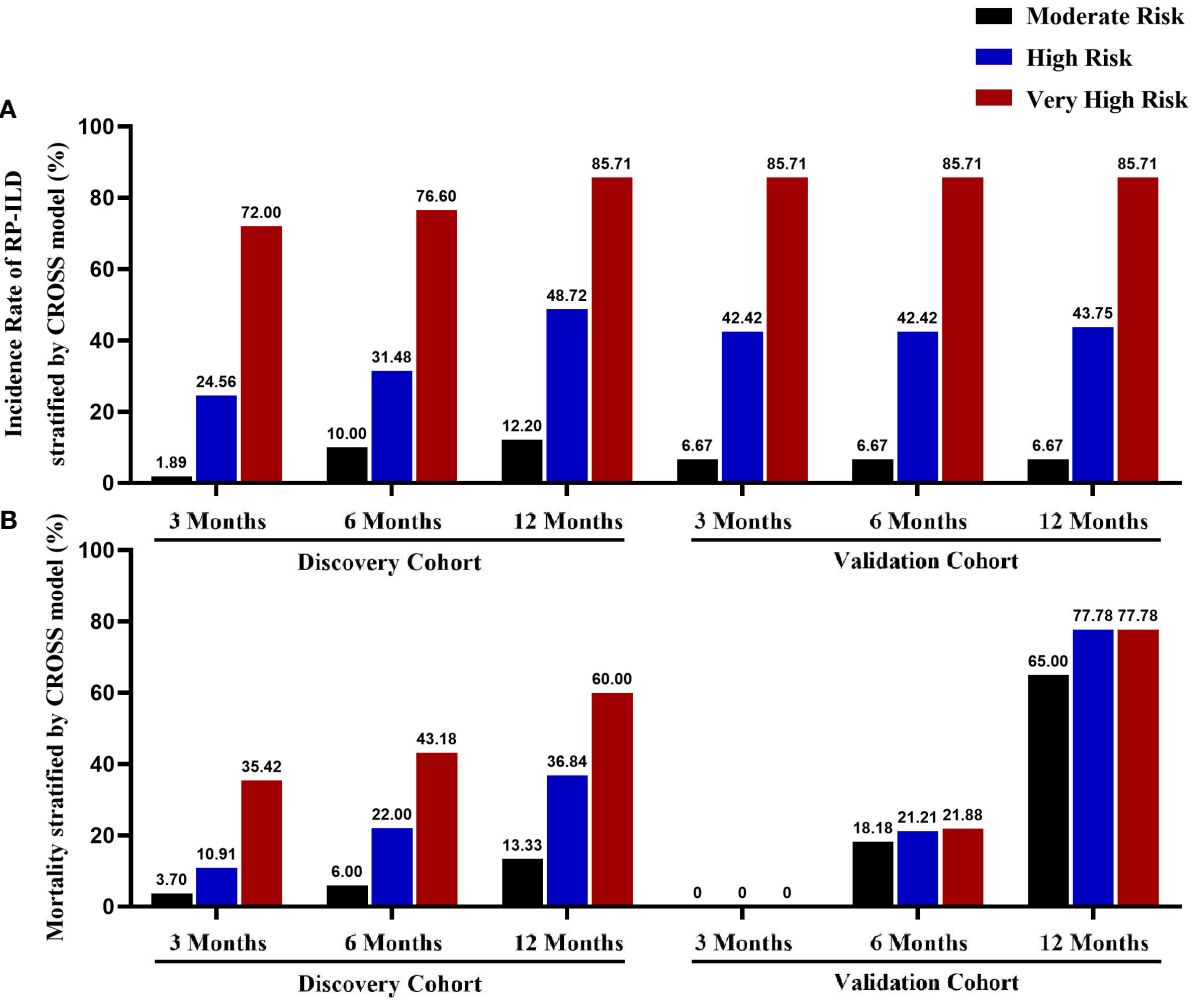
The incidence rate of RP-ILD and mortality according to the CROSS model Comparison of 3-months, 6-months, and 1-year incidence rates of RP-ILD **(A)** and mortality **(B)** in the discovery and validation cohorts with different risk stratification.

At last, we calculated the rates of RP-ILD in anti-MDA5^+^DM patients with moderate, high, and very high-risk stratification at different time points. As the risk level rises, the incidence rate of RP-ILD based on the CROSS model was gradually raised in both cohorts. Of note, the incidence rate of RP-ILD in very high-risk patients is over 70%, high-risk patients are around 25%-50%, and less than 15% in moderate-risk patients (**Figure 4**).

## 4 Discussion

RP-ILD is a common and potentially fatal complication of anti-MDA5^+^DM. Risk stratification to predict patients who will develop fatal RP-ILD at the early stage of the disease is very important for discussing patient expectations and supporting therapy decision-making in anti-MDA5^+^DM patients. Based on the current largest reported anti-MDA5^+^DM cohort containing 255 consecutive anti-MDA5^+^DM patients, we developed and validated a CROSS model that successfully predicts RP-ILD and mortality risk.

There are three essential differences between the FLAIR model (11) mentioned in introduction and our own. First, all patients included in our cohort are anti-MDA5^+^DM patients. Second, unlike the FLAIR model for predicting death risk, the CROSS model represents the first prognostic tool for RP-ILD in anti-MDA5^+^DM patients. RP-ILD is linked to high mortality. Given that only about 1/3 of anti-MDA5^+^DM may develop RP-ILD after disease onset in our cohort and the previous report, early recognition of patients with high RP-ILD risk is particularly important in halting disease progression and improving prognosis in anti-MDA5^+^DM. Third, the inconvenience of obtaining HRCT scoring and anti-MDA5 titers data limits the FLAIR model’s widespread adoption in daily clinical practice (12–14). To overcome these limitations, we developed a simpler prognostic model.

We compared 26 clinical or laboratory parameters from 255 patients (94 RP-ILD and 161 non-RP-ILD; 192 survivors and 63 non-survivors). Amongst all parameters investigated, we found four parameters to be independent risk factors for RP-ILD in the discovery cohort. We then developed and externally validated a CROSS (CRP, anti-Ro52 antibody, short disease duration, Sex[male sex]) for predicting RP-ILD risk in two different anti-MDA5^+^DM cohorts. The CROSS model is an easy-to-use risk prediction system based on commonly used and easily obtained variables in the clinical setting. In both discovery and validation cohorts, very high-risk patients had significantly higher RP-ILD rates than moderate- and high-risk patients. Interestingly, the risk of death in anti-MDA5^+^DM patients was also well stratified based on the predictive model (**Figures 3C, D**). However, there was no significant difference in overall mortality among the three groups based on the CROSS model in the validation cohort (**Figure 4B**).

According to the CROSS model, 51 patients (30.0%) were classified as at very high risk of developing RP-ILD in the discovery cohort, and 76.6% of them eventually developed RP-ILD in the first 6 months. Similar accuracy was confirmed in the validation cohort and achieved 85.7%. Stratifying anti-MDA5^+^DM into different risk categories allows for closer monitoring of very high-risk patients and guiding management decisions.

In our cohort, 34.84% of anti-MDA5^+^DM patients will develop RP-ILD during the first 3 months after disease onset. Actually we find significant, time- time-dependent changes in RP-ILD and mortality risk in MDA5+ DM patients in our cohort. More than 90% RP-ILD and 84% mortality occurs in the first 6-months after disease onset. Notably, the first 3-months is a particularly high-risk period, with 50% RP-ILD and 46% death occurring. We proposed the first 6-months, especially the first 3-months, is a risk window for the poor outcome in anti-MDA5+ DM patients (19). We recommend calculating the CROSS score initially after the patient is diagnosed with anti-MDA5^+^DM. Then, the CROSS risk classification system can be used sequentially, particularly in the first 3~6 months.

Among the variables in the CROSS model, the increased CRP levels imply high disease activity and hyperinflammatory state in anti-MDA5^+^DM. We previously identified 3 distinct phenotypes with significantly different prognoses in patients with anti-MDA5^+^DM. The most prominent feature in anti-MDA5^+^DM with RP-ILD is the high inflammatory status (20), supporting CRP played an important role in the CROSS score. CRP is a dynamic index. Besides its association with an inflammatory state, CRP levels are also linked to potential infection, which might warn differential diagnosis during disease progression. Based on the requirement of simplification of the clinical model, all quantitative parameters were qualified based on whether they were abnormal, which may even weaken the impact of CRP in predicting the prognosis of anti-MDA5^+^DM patients.

In our CROSS model, anti-Ro52 is a strong prognostic factor for RP-ILD. Anti-Ro52 is the most common autoantibody detected in polymyositis and the anti-synthetase syndrome. Previous studies have reported that anti-Ro52 antibodies significantly correlated with ILD in DM, juvenile myositis, primary Sjögren syndrome, and connective tissue diseases, suggesting anti-Ro52 is an intended risk factor for ILD (21–25). Consistent with our findings, recent studies have found that the coexistence of anti-MDA5 and anti-Ro52 correlates with an increased frequency of RP-ILD and poor prognosis in anti-MDA5 DM patients (25). Thus, the presence of anti-Ro52 might help to distinguish a subgroup of anti-MDA5^+^DM patients with more aggressive phenotypes.

There are limitations to this study. First, all data are obtained from a multicenter retrospective study, and missing data could not be avoided, which might be a bias of the analysis. Second, FVC, DLCO values, and hypoxemia have been reported as risk factors for RP-ILD and poor prognosis in patients with anti-MDA5^+^DM (7). Approximately36.9% of patients develop RP-ILD during the follow-up period in our cohort, and most patients have no markedly respiratory symptoms at the beginning of disease onset. Our goal is to create an easy-to-use prediction model that could be applied at the initial visit of a patient with anti-MDA5 DM. Therefore, we did not include lung function and arterial blood gas in our risk classification system. Third, the treatment regimen was not analyzed in the current study, especially lacking the relationship between changes in CROSS score over time with treatment response. Fourth, the worldwide coronavirus disease 2019 (COVID-19) pandemic indicated a similar cytokine storm in anti-MDA5^+^DM with RP-ILD. CROSS score did not take into account blood cytokine profiles with clinical outcomes. Given that the development and validation of the CROSS model were conducted with patients from various hospitals in Jiangsu Province, China, its applicability to populations in other regions or ethnic groups beyond Asians has yet to be established. This necessitates additional validation to confirm its effectiveness in diverse settings. Despite its limitations, based on the most significant reported study populations, our CROSS score provides a simple and accurate model for predicting RP-ILD onset and mortality risk in anti-MDA5^+^DM patients. Further prospective studies are needed to validate its accuracy in risk prediction further, facilitating the truly clinical decision-making support in anti-MDA5^+^DM patients.

Recently, Jacqueline So et al. also revealed a FLAW model that based on fever, LDH, age, and white cell count maybe useful to predict the risk of RP-ILD in anti-MDA5^+^DM patients (26). Similar to our CROSS model, FLAW provide a simple pragmatic model for predicting RP-ILD. Although the variables are different, both these two models showed good predictive effects. Future prospective studies are needed to compare the clinical application value of these two models. Using the largest reported cohort, we developed and validated a prediction model for RP-ILD risk in anti-MDA5^+^DM patients. The strength of this model is the use of clinical variables that could be easily obtained during the routine clinic visit. This simple predictive model could aid in the early detection of anti-MDA5^+^DM patients without RP-ILD at poor prognosis risk, guiding treatment and improving outcomes.

## 5 Conclusions

The CROSS model could help to identify anti-MDA5^+^DM patient who are at high risk of RP-ILD. It provides a simple and easy-used mothed to early warning and early detection of anti-MDA5^+^DM patients with poor outcomes in in daily clinical practice.

## Data availability statement

The raw data supporting the conclusions of this article will be made available by the authors, without undue reservation.

## Ethics statement

The studies involving humans were approved by the ethical committee of the First Affiliated Hospital of Nanjing Medical University: 2020-SR-265. The studies were conducted in accordance with the local legislation and institutional requirements. The participants provided their written informed consent to participate in this study. Written informed consent was obtained from the individual(s) for the publication of any potentially identifiable images or data included in this article.

## Author contributions

LW: Conceptualization, Data curation, Investigation, Writing – original draft, Formal Analysis, Methodology, Project administration, Software, Visualization. CL: Conceptualization, Supervision, Validation, Writing – review & editing. HY: Writing – review & editing, Data curation, Supervision, Validation. LX: Data curation, Writing – review & editing, Supervision, Validation. FY: Writing – review & editing, Investigation. JLi: Investigation, Writing – review & editing. MW: Investigation, Writing – review & editing. SZ: Investigation, Writing – review & editing. ZD: Investigation, Writing – review & editing. JQ: Investigation, Writing – review & editing. HW: Investigation, Writing – review & editing. WY: Investigation, Writing – review & editing. LZ: Investigation, Writing – review & editing. YW: Investigation, Writing – review & editing. SY: Investigation, Writing – review & editing. DZ: Investigation, Writing – review & editing. JW: Investigation, Writing – review & editing. YL: Investigation, Writing – review & editing. DS: Investigation, Writing – review & editing. ZL: Investigation, Writing – review & editing. LL: Investigation, Writing – review & editing. LM: Investigation, Writing – review & editing. XX: Investigation, Writing – review & editing. YZ: Investigation, Writing – review & editing. HL: Investigation, Writing – review & editing. TR: Investigation, Writing – review & editing. JLiu: Writing – review & editing, Data curation. FW: Writing – review & editing, Funding acquisition. MZ: Writing – review & editing, Data curation. WT: Data curation, Conceptualization, Funding acquisition, Investigation, Supervision, Writing – original draft.

## Supplementary material

Supplementary Figure 1 A nomogram predicting RP-ILD risk of anti-MDA5^+^DM patients Each variable’s value was given a score on the point scale axis. A total score could be calculated by adding every single score, and by projecting the complete score to the lower full-point scale, we could estimate the probability of RP-ILD.

Supplementary Figure 2 Differences in CROSS scores in anti-MDA5^+^DM patients The violin diagram shows the difference in CROSS scores between patients who progressed to RP-ILD and those who did not in both discovery **(A)** and validation cohort **(B)**.

Supplementary Figure 3 AUC value of time-dependent ROC curve in CROSS model The area under the curve (AUC) value of the time-dependent ROC curve shows that the CROSS model has excellent differentiation efficiency in both cohorts within different time points.
