# Supplementary figures and images for: Rapidly progressive interstitial lung disease risk prediction in anti-MDA5 positive dermatomyositis: the CROSS model
Source: Front Immunol. 2024 Feb 1;15:1286973. doi: 10.3389/fimmu.2024.1286973 (PMC10867574; doi:10.3389/fimmu.2024.1286973)

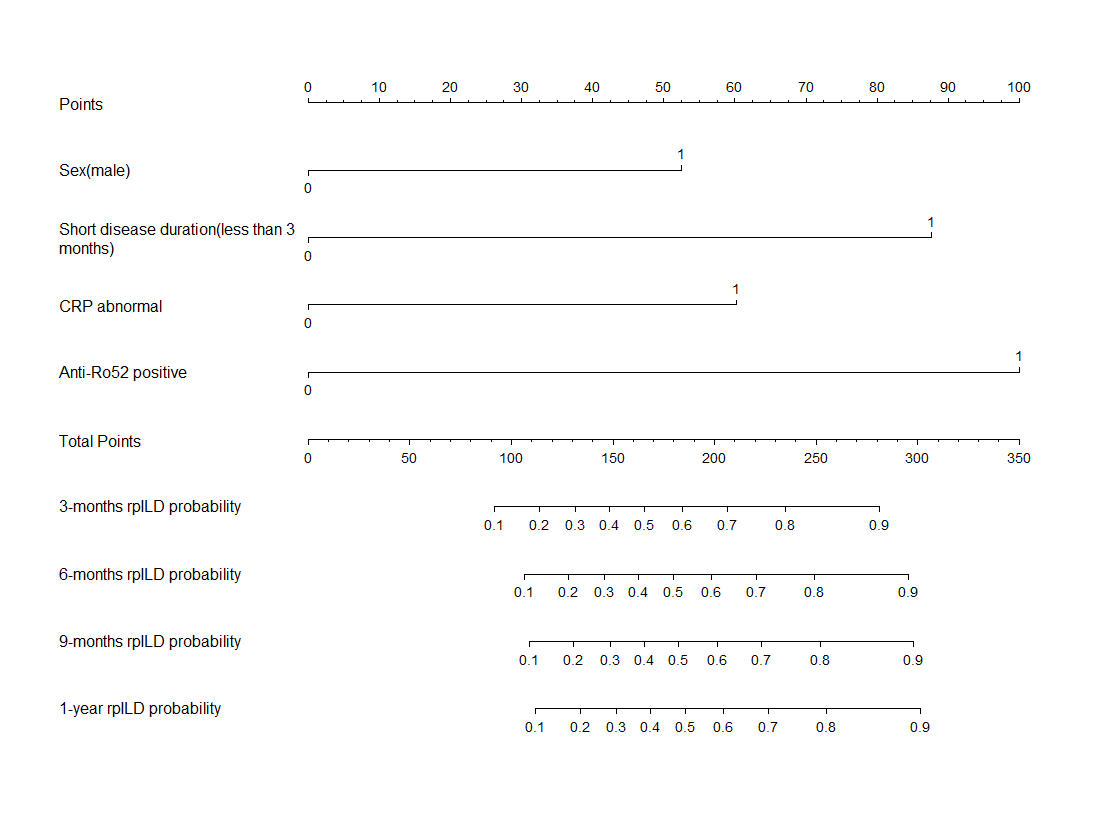

Supplement: Supplementary Figure 1 — A nomogram predicting RP-ILD risk of anti-MDA5+DM patients Each variable’s value was given a score on the point scale axis. A total score could be calculated by adding every single score, and by projecting the complete score to the lower full-point scale, we could estimate the probability of RP-ILD. [file Image_1.tiff]

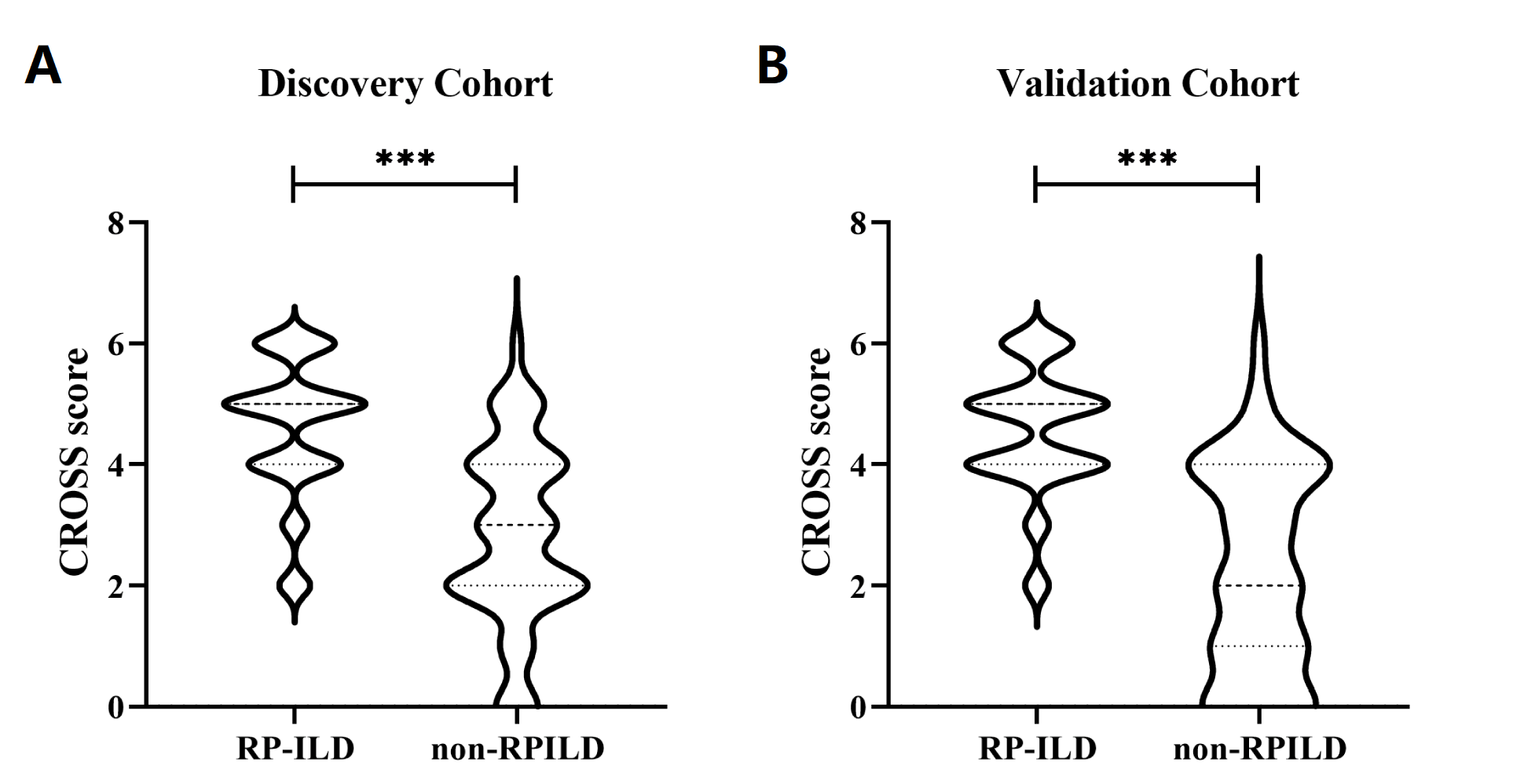

Supplement: Supplementary Figure 2 — Differences in CROSS scores in anti-MDA5+DM patients The violin diagram shows the difference in CROSS scores between patients who progressed to RP-ILD and those who did not in both discovery (A) and validation cohort (B). [file Image_2.tif]

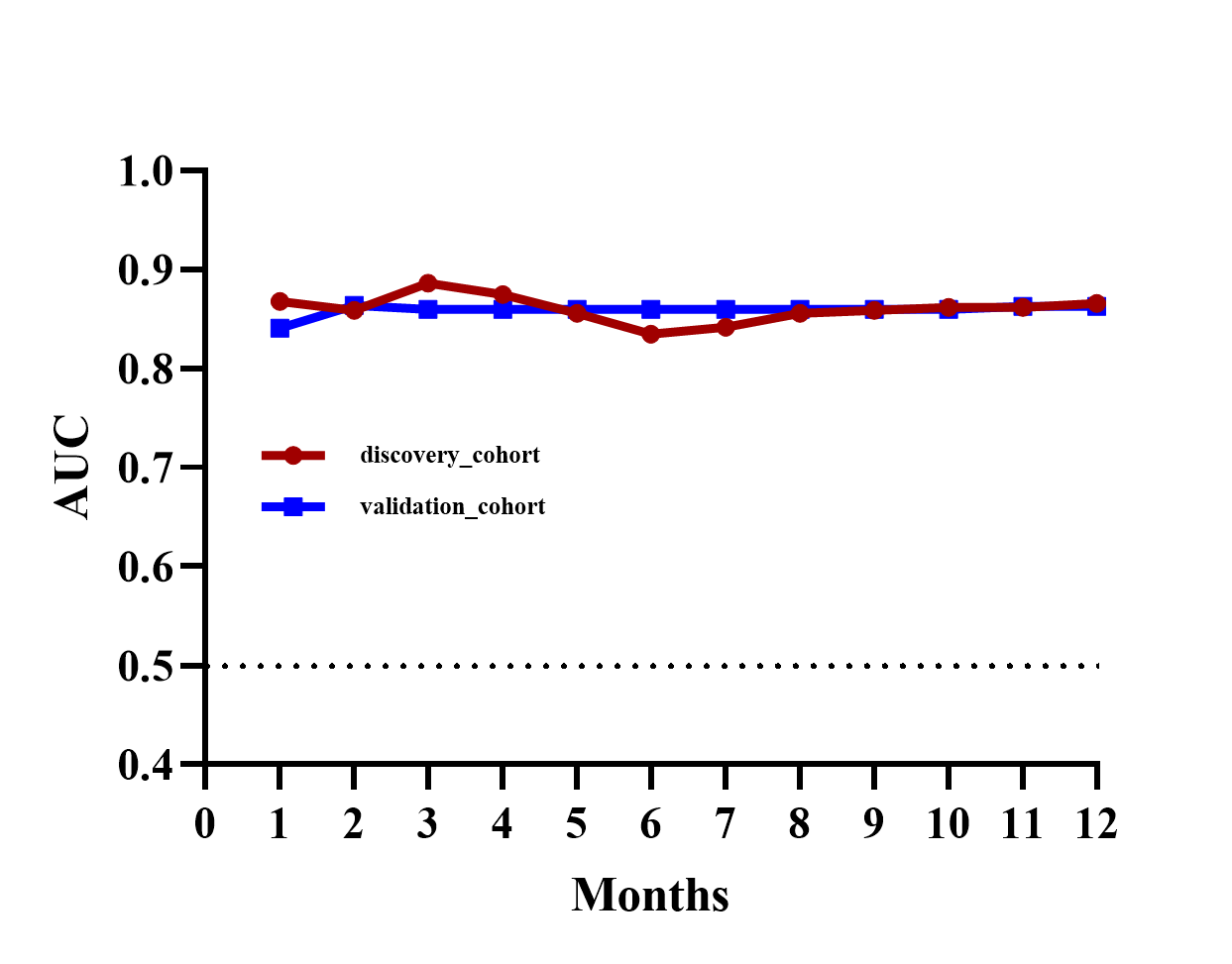

Supplement: Supplementary Figure 3 — AUC value of time-dependent ROC curve in CROSS model The area under the curve (AUC) value of the time-dependent ROC curve shows that the CROSS model has excellent differentiation efficiency in both cohorts within different time points. [file Image_3.tif]
